# Relapsing *Plasmodium vivax* malaria in a 12-year-old Brazilian girl: A case report

**DOI:** 10.5281/zenodo.11125657

**Published:** 2024-05-07

**Authors:** Ezequias B. Martins, Anielle de Pina-Costa, Roxana F. Mamani, Otilia Lupi, Guilherme A. Calvet, Clarisse S. Bressan, Michele F. B. Silva, André M. Siqueira, Sidnei da Silva, Graziela Maria Zanini, Maria de Fátima Ferreira-da-Cruz, Cláudio Tadeu Daniel-Ribeiro, Patrícia Brasil

**Affiliations:** 1 Instituto Nacional de Infectologia Evandro Chagas, Fundação Oswaldo Cruz (Fiocruz), Rio de Janeiro, Brazil.; 2 Centro de Pesquisa, Diagnóstico e Treinamento em Malária (CPD-Mal) da Fiocruz e da Secretaria de Vigilância em Saúde e Ambiente, Ministério da Saúde, Brazil.; 3 Laboratório de Pesquisa em Malária, Instituto Oswaldo Cruz, Fiocruz, Rio de Janeiro, Brazil.

## Abstract

*Plasmodium vivax* causes the vast majority of malaria cases in Brazil. The lifecycle of this parasite includes a latent stage in the liver, the hypnozoite. Reactivation of hypnozoites induces repeated relapses. We report a case of two relapses of *vivax* malaria in a teenage girl after conventional treatment with chloroquine and primaquine. Chloroquine prophylactic treatment for three months was prescribed with a favourable outcome of the case.

## Introduction

Malaria remains a major global public health problem, with a substantial increase in cases reported in the Americas in recent years, mainly in Brazil and Venezuela [1,2]. In the Brazilian Amazon, most malaria cases are caused by *Plasmodium vivax*, a parasite that causes less severe disease compared to *P. falciparum*, but its biological characteristics present major challenges for current infection treatment and cure strategies [3,4].

In the last decades, the treatment of malaria made significant advances, providing a marked decrease in mortality worldwide. However, the elimination of *P. vivax* is hampered due to malaria relapses by activating hypnozoites, a population of sporozoites that undergo dormancy in hepatocytes. Hypnozoites are carried silently, with no symptoms. Reactivation of *P. vivax* hypnozoites from the dormant stage of the parasite causes clinical relapses. Hypnozoites causing relapses may be reactivated in as short as two weeks or as long as 10 months after the initial infection [5]. In tropical climates, relapses occur at short intervals (3-4 weeks after treatment) [6].

The main factors involved in relapses are non-adherence to treatment, parasite resistance to drugs, poor drug quality or sub-therapeutic drug doses [7]. Primaquine is the drug of choice for eliminating the hypnozoite form of the parasite [8]. Until recently, primaquine was the only option for *P. vivax* liver-stage treatment. In 2018, following the regulatory approval of tafenoquine (a primaquine analogue with a half-life of approximately 15 days), this drug became an interesting alternative, especially because of the single-dose regimen reducing the risk of low or non-compliance [9,10]. Both drugs are 8-aminoquinolines and need to be given with an appropriate blood stage drug to achieve radical cure [11,12].

## Case Report

A healthy 12-year-old female Brazilian patient was referred to the the Acute Febrile Diseases Reference Center (CPD-Mal) at the Instituto Nacional de Infectologia Evandro Chagas, Fiocruz (Rio de Janeiro, Brazil) with high fever, chills, severe headache, and mild diarrhoea. The patient reported having been diagnosed with *P. vivax* malaria (15 parasites/mm^3^) on January 15th, 2021 in the local Reference Center during a consultation when she was living in the Brazilian Amazon. She was treated with a chloroquine and primaquine regimen, with correct drug dosage and treatment time.

On May 13^th^, 2021 (4 months after the first infection), a recurrence of *P. vivax* was diagnosed using a species-specific real time polymerase chain reaction (PCR) capable to detect submicroscopic parasitemias [13], as well as Abbot Pf/Pf/Pv RDT^®^ and thick blood smear (TBS); no *P. falciparum* or *P. malariae* mixed infections were detected by PCR [14,15]. A new treatment with chloroquine and primaquine was prescribed according to the Brazilian treatment guidelines. The same malaria tests (PCR, RDT^®^ and TBS) were negative after this course of treatment. This new episode was considered a malaria relapse as the patient did not return to a malaria-endemic area and there was no epidemiological history to support the possibility of a new infection.

In July 2021 (6 months after the first infection), the patient developed high fever, chills, asthenia, and dyspnea. Again, the same PCR, RDT^®^ and TBS tests diagnosed a new relapse of *P. vivax* infection. Treatment with artesunate and mefloquine was recommended for this second relapse. The patient required hospitalisation and evolved with thrombocytopenia without bleeding episodes. Laboratory tests confirmed clinical cure after the therapeutic regimen. The patient was hospitalised for four days during the second recurrence that did not progress to clinical severity. She was discharged from the hospital with clinical and parasitological cure attested by parasite clearance through PCR, RDT^®^ and TBS clearance on July 29^th^, 2021. In this second relapse, the patient had a much greater parasitaemia, predisposing to greater destruction of red blood cells, causing congestion in the spleen and producing platelet sequestration, justifying the transient thrombocytopenia. Table 1 shows the main laboratory tests performed during first and second relapses follow-up.

**Table 1.** Laboratory tests and outcomes performed during *P. vivax* relapses in a 12-year-old girl from Brazil.

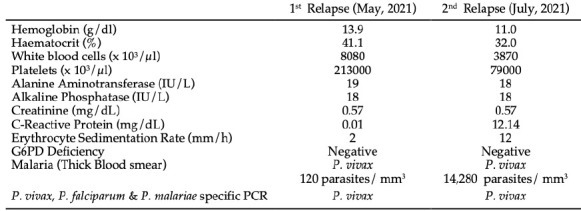

Subsequently, the patient received prophylactic treatment with chloroquine weekly for three months (July to October 2021) and after twelve months no malaria recurrence was detected by PCR, RDT^®^ and TBS. Table 2 shows the therapeutic regimens used during follow-up.

**Table 2.** Therapeutic regimens used during recurrent *P. vivax* infections in a 12-year-old girl (bodyweight 49 kg) from Brazil.

Dates	Drug	Dose (mg)	Regimen	Duration
January 2021	Chloroquine	450	Once/day	3 days
	Primaquine	30	Once/day	7 days
May 2021	Chloroquine	450	Once/day	3 days
	Primaquine	30	Once/day	7 days
July 2021	Artesunate	200	Once/day	3 days
	Mefloquine	400	Once/day	3 days
July-October 2021	Chloroquine	225	Once/week	12 weeks

## Discussion

This case report demonstrated that infections with Brazilian isolates of *P. vivax* can relapse, despite adequate use of currently indicated drugs. Two relapses were documented within six months, raising doubts about the effectiveness of primaquine in eliminating the hypnozoite forms of the parasite (radical cure). Pina-Costa *et al.* [16] reported a series of three cases of malaria relapses, where an increase in primaquine dosage was attempted, but only two patients had satisfactory results. For the patient who did not respond to high doses of primaquine, prophylactic treatment with chloroquine (one weekly dose) was performed for three months, with radical cure. In our case, we tested Pina-Costa *et al.*'s recommendation and used chloroquine weekly for three months, achieving a radical cure of the parasite.

Chloroquine, artesunate, and mefloquine are effective drugs for treatment of blood stage *P. vivax* infections, and primaquine is routinely used for radical cure of hypnozoites [17]. Achieving radical cure is the most significant therapeutic challenge in *vivax* malaria.

When Glucose-6-Phosphate Dehydrogenase (G6PD) status is normal, primaquine use is the gold standard of care [5]. Our patient used the medication regularly, with correct doses, and did not have G6PD deficiency. Since tafenoquine can only be prescribed for patients over 16 years old, even if the medicine would have been available, it was not eligible for the 12-year-old patient treatment. In this way, the treatment with primaquine was the only feasible option for managing the case.

## Conclusion

A multitude of factors play a role in *P. vivax* recurrences, such as patient adherence to treatment, flawed treatment protocols, inadequate metabolism of primaquine, *Plasmodium* resistance to anti-malarial medications, and besides unknown factors. Managing cases of *vivax* malaria remains a complex challenge, necessitating healthcare professionals to closely adhere to guidelines in order to enhance treatment outcomes and reduce the impact/transmission of the disease.
